# **Astrocytes as Perspective Targets of Exercise- and Caloric Restriction‐Mimetics**

**DOI:** 10.1007/s11064-021-03277-2

**Published:** 2021-03-07

**Authors:** Ulyana Lalo, Yuriy Pankratov

**Affiliations:** 1 School of Life Sciences, Immanuel Kant Baltic Federal University, Kaliningrad, Russia; 2 School of Life Sciences, University of Warwick, Coventry, UK

**Keywords:** Caloric restriction, Exercise, Synaptic plasticity, Autophagy, Spemidine, Beclin 1, Glia-neuron interactions, Mitochondrial calcium, Aging

## Abstract

Enhanced mental and physical activity can have positive effects on the function of aging brain, both in the experimental animals and human patients, although cellular mechanisms underlying these effects are currently unclear. There is a growing evidence that pre-clinical stage of many neurodegenerative diseases involves changes in interactions between astrocytes and neurons. Conversely, astrocytes are strategically positioned to mediate the positive influence of physical activity and diet on neuronal function. Thus, development of therapeutic agents which could improve the astroglia-neuron communications in ageing brain is of crucial importance. Recent advances in studies of cellular mechanisms of brain longevity suggest that astrocyte-neuron communications have a vital role in the beneficial effects of caloric restriction, physical exercise and their pharmacological mimetics on synaptic homeostasis and cognitive function. In particular, our recent data indicate that noradrenaline uptake inhibitor atomoxetine can enhance astrocytic Ca^2+^-signaling and astroglia-driven modulation of synaptic plasticity. Similar effects were exhibited by caloric restriction-mimetics metformin and resveratrol. The emerged data also suggest that astrocytes could be involved in the modulatory action of caloric restriction and its mimetics on neuronal autophagy. Still, the efficiency of astrocyte-targeting compounds in preventing age-related cognitive decline is yet to be fully explored, in particular in the animal models of neurodegenerative diseases and autophagy impairment.

## Introduction

Due to changing demographics, age-related cognitive decline and neurological disorders have become a real medical and societal problem. Maintaining of cognitive function during ageing as well as adaptation of brain to environmental, metabolic and immunological challenges relies on synaptic plasticity-an active, continuous process occurring throughout the lifespan [1–4]. Remarkable responsiveness of brain cellular networks and synapses to environmental enrichment, physical activity and changes in the diet can help to alleviate the negative impact of ageing on cognitive function [2, 4–6]. Recent data [5, 7] show that environmental enrichment (EE) and caloric restriction (CR) can ameliorate the aging-related alterations in synaptic transmission and mitigate the age-related decline in synaptic plasticity in the neocortical neurons.

A growing evidence of beneficial effects of exercise and diet on brain function gave rise to a concept of “cognitive reserve”-a set of cellular and molecular mechanisms which allow the brain to adapt to age-related pathologies despite underlying neural changes [8]. This concept is particularly attractive in view of necessity to develop pharmaceutical agents for prevention/amelioration of cognitive decline [3, 8–11]. Since many elderly, apparently healthy, individuals cannot endure prolonged diets or vigorous exercise due to pre-diabetes or other symptoms of imminent disease, usage of pharmacological agents which mimic the beneficial effects of exercise and CR without provoking their discomfort is attracting an increasing interest [2, 3, 8, 9, 12, 13]. Progress in the development of such pharmaceuticals, commonly referred as *“exercise-mimetics“ or “CR-mimetics*”, is hampered by the gaps in our understanding of cellular mechanisms of brain longevity and cognitive reserve. Numerous recent studies suggest that neuro-centric view on brain aging and neurodegenerative diseases is not compatible with clinical observations, so it becomes evident that brain pathologies can be fully understood only in the context of the complex cellular networks responsible for the brain homeostasis, i.e., astrocyte-neuron, astrocyte-microglia and neurovascular units [14–18].

In this article we overview the emerging evidence of importance for astrocytes in the mechanisms of cognitive reserve which render astroglia a perspective target for CR- and exercise mimetics.

## Role for Astroglia in the Mechanisms of Cognitive Reserve

The results of studies carried out over the last three decades show that astrocyte have an active role not only in the metabolic support of neurons but also in the signal processing in the neural networks [14, 18–20]. Although electrical signalling is quintessential for the brain function, neurons working alone seem to provide only a partial explanation for the mechanisms of brain plasticity, homeostasis and longevity [14, 16–18]. Numerous studies demonstrated that astrocytes are chemically excitable and express a plethora of receptors, in particular to glutamate, noradrenaline, ATP and endocannabinoids [5, 7, 21–23]. These receptors enable astroglia to respond to transmitters spilled out of nearby synapses with an elevation of the cytosolic Ca^2+^-level [19–24]. In return, astrocytes respond to the neurons by releasing small molecule transmitters, such as ATP, glutamate and BDNF, which modulate synaptic transmission and plasticity [5, 14, 19, 25–28]. Hence, being in bi-directional communication with neurons, astrocytes can indeed play an active roles in storage and processing of information in the brain[14, 20]. Also, astrocytes mediate metabolic coupling between brain vasculature and neurons [20, 29] and thereby are ideally placed to couple effects of physical activity and diet to the wellbeing of neurons.

Recent research highlighted the need for greater mechanistic understanding of neuron-glia interactions in aging and late-life diseases[15, 16, 18, 30, 31]. There is accumulating evidence that functional changes in glia can precede molecular and functions changes in neurons and neurodegeneration[16, 17, 31, 32]. Astrocytes are plastic and can undergo complex morphological, molecular and functional alterations during physiological ageing and in neurodegenerative diseases; these alterations range from the loss of functions (atrophy) to reactive astrogliosis (hypertrophy) [5, 7, 17, 31, 33–35]. In particular, Ca^2+^ signalling and release of gliotransmitters from neocortical astrocytes can undergo significant age-related decline [6, 7, 33]. On another hand, astrocytic functions and astroglia-neuron interactions can enhanced by physical activity, environmental enrichment and changes in a diet [5–7, 17, 36, 37]. In particular, the EE and CR were reported to increase the astrocytic Ca^2+^ signalling and release of gliotransmitters in the neocortex of aged mice, which was accompanied by alterations in synaptic transmission and plasticity [5, 7, 28]. Furthermore, our data demonstrate that EE- and CR-induced enhancement of synaptic plasticity and memory are inhibited in the dnSNARE transgenic mice[5, 7, 34] with impaired exocytosis of gliotransmitters suggesting an important role for astrocytes in cognitive benefits of EE and CR.

Thus, astroglia and efficient astrocyte-neuron interactions are crucial component of brain’s cognitive reserve. The age-related impairment of capability of astrocytes to receive and integrate signals from neurons may compromise astroglial modulation of synaptic transmission and therefore lead to a decline in cognitive function. Conversely, an active lifestyle and caloric restriction can enhance astrocytic regulation of neuronal function and thereby prevent or even cure the age-related cognitive impairment. Yet, it remains to be determined how pharmacological manipulation of astrocytes can prevent specific neurological disease initiation or ameliorate progression of cognitive decline.

## Astroglial Adrenergic Glial Signalling as Potential Target of Exercise‐Mimetics

An active lifestyle and physical exercise in particular are commonly believed to benefit both physical and mental health [2, 4]. Indeed, numerous studies reported beneficial effects of active lifestyle and its proxy in animal experiments – environmental enrichment - on function of aging brain, both in human patients and rodent models [4, 10–12]. It has been shown that physical exercise (PE) and environmental enrichment (EE), which in rodent experiments conventionally includes *ad libitum* exercise, can reverse atrophic changes in neurons [1, 2] and avert the decline in the synaptic plasticity and memory associated with aging and neurodegenerative diseases [4, 5, 7, 10–12]. However, exercise as therapeutic intervention is not easily applicable for diseased or elderly patients, especially in current conditions of self-isolation during pandemic diseases. Thus, development of exogenous pharmacological agents capable to activate cellular targets of exercise and reproduce its cognitive benefits (“exercise-mimetics”) is viewed as a vital task [2, 38, 39].

The majority of recent studies of potential exercise-mimetics have been focused mainly on metabolic factors activated in skeletal muscles during exercise, such as 5’ adenosine monophosphate-activated protein kinase (AMPK) and peroxisome proliferator-activated receptor δ (PPARδ) which have been reported to have some beneficial cognitive effects [40, 41]. It was suggested that effects of these factors are mediated by molecular cascades involving the sirtuins (e.g. SIRT1) and PPARδ coactivator 1α (PGC-1α) [12, 40, 41], so the activator of AMPK, AICAR and exogenous agonist of PPARδ, GW501516, have emerged as perspective exercise-mimetics [38, 40]. One should note that majority of molecular interactions of AMPK, SIRT1 and PGC-1α have been linked to the caloric restriction modulation of autophagy[9, 42] (discussed below); the specific effects of these proteins in astrocytes are not well-studied. Most importantly, the efficiency of AICAR and GW501516 in human patients, even in reproducing cardiovascular benefits of PE, has been put under scrutiny [39, 43] so candidate substances to reproduce cognitive benefits of exercise should be sought elsewhere.

The noradrenergic neuromodulatory system has recently emerged as an important component of cognitive reserve [8, 44, 45]. In particular, increase in the brain noradrenaline (NA) concentration during physical exercise and mental activity have been shown to mitigate the negative impact of aging and neurodegeneration on synaptic plasticity, memory and cognitive flexibility [44–46]. Clinical studies on usage of adrenoreceptor agonists and noradrenaline (NA) uptake inhibitors for treatment of age- and AD-related depression are gaining momentum [8, 44, 47, 48]. Many of NA uptake inhibitors permeate the brain-blood barrier, are orally-active and have attracted a significant attention as “cognitive enhancers”[47, 48].

Interestingly, the release of noradrenaline in the brain cortex occurs via “volume transmission”, i.e. from varicosities of adrenergic neurons into brain extracellular fluid, rather than into a cleft of specialized synapses [44, 48]. Hence, astrocytes can be the first cells to respond to the diffuse NA-mediated signals and transmit the information to neurons. Indeed, adrenergic signaling has been reported to modulate the activity of astrocyte networks according to the behavioral state or sensory inputs [14, 15]. Astrocytes abundantly express adrenergic α1-receptors [44, 49] which have been demonstrated to activate Ca^2+^-dependent release of gliotransmitters and mediate astroglia-driven modulation of synaptic plasticity [5, 22, 50]. Furthermore, adrenergic signalling in astrocytes can increase during physical activity or after environmental enrichment [5, 36, 49]. So, astroglial adrenoreceptors, in particular α1-ARs, represent an important part of a “cognitive reserve” and perspective target for adrenergic modulators.

**Fig. 1:**
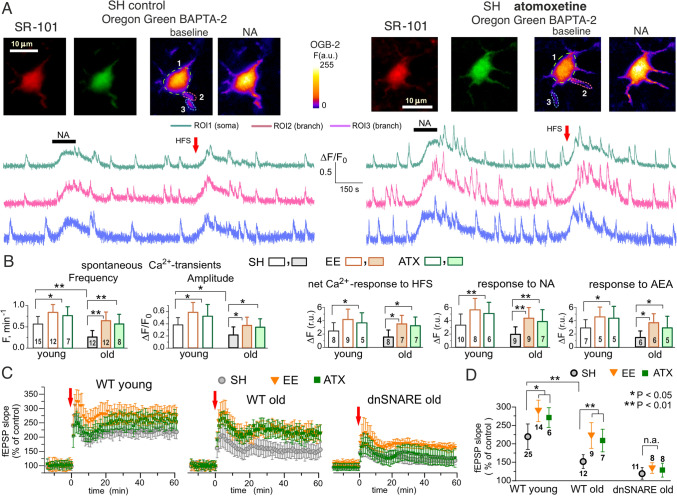
Effect of atomoxetine on the astroglial Ca^2+^ signaling and synaptic plasticity in the hippocampus. **a**, **b** Ca^2+^-signalling was evaluated in the astrocytes of CA1 area of hippocampal slices, as described previously [5, 22, 25]. The 2–5 month-old (young) and 14–18 month-old mice (old) were kept either under standard housing conditions (SH) or exposed to the enriched environment (EE). Some hippocampal slices of SH mice were incubated with 200 nM NA-uptake inhibitor atomoxetine for 2 hrs followed by 30 min washout prior to recordings (ATX). **a** Representative multi-photon images of astrocytes of old mice pre-loaded with Ca^2+^-probe OregonGreen BAPTA-2 AM and stained with fluorescent astroglial marker SR101 and presudo-color images of OGB-2 fluorescence recorded before and after application of noradrenaline (NA, 1 µM). Graphs below show the time course of OGB-2 fluorescence averaged over regions indicated in the fluorescent images before and application of NA and after the 100 ms-long episode of 100 Hz high-frequency stimulation (HFS) of Schaffer collaterals. **b** The pooled data (mean ± SD for the number of cells indicated) on the peak amplitude and frequency of the baseline spontaneous Ca^2+^-transients and the net responses to the HFS, NA and endocannabinoid receptors agonist AEA (500 nM) evaluated in the astrocytes of different age and treatment groups as described in [5, 28]. Asterisks (*, **) indicate statistical significance (unpaired t-test) of the effect of age, EE- and atomoxetine-treatment. Note the significant decrease in the spontaneous and evoked astroglial Ca^2+^-signalling and increase induced by EE and atomoxetine. Note the significant decrease in the spontaneous and evoked astroglial Ca^2+^-signalling and increase induced by EE and atomoxetine. **c**, **d** Long-term potentiation (LTP) of the field EPSPs recorded in the hippocampal CA1 area (*stratum radiatum*) of wild-type and dnSNARE mice with impaired gliotransmission [5, 25]. **c** The time course of changes in the fEPSP induced by 1 s-long HFS delivered at zero time, measured in the WT and dnSNARE mice of the same age and treatment groups as in (**a**, **b**). Dots in the graphs represent the average of 6 consecutive fEPSPs; data are shown as mean ± SD for number of experiments indicated in the panel (D). Data were normalized to the fEPSP slope averaged over 10 min period prior to the HFS. **d** The pooled data on the magnitude of LTP ( mean ± SD) evaluated as relative increase in the fEPSP slope at 60th min, averaged across 10 min time window. Note the significant decrease in the LTP magnitude in old mice and significant effects of EE and atomoxetine in the wild-type but not the dnSNARE mice

It is conceivable that pharmacological modulation of noradrenergic signalling by the NA re-uptake inhibitors could recapitulate effects of physical exercise on glia-neuron communication, synaptic transmission and cognitive functions. In line with this notion, our recent experiments showed that long-term incubation of hippocampal slices of old mice with small concentrations of atomoxetine (200 nM) enhanced the astrocytic Ca^2+^-signalling (Fig. 1a, b) and significantly increased the magnitude of long-term synaptic potentiation (LTP) in the CA1 area, thereby recapitulating the beneficial effects of EE (Fig. 1c, d). Atomoxetine (ATX) has much higher affinity to the NET/SLC6A2 noradrenaline transporters and, in the concentration used in our experiments, does not affect uptake of dopamine or serotonin via DAT and SERT transporters [51]. Since SLC6A2 transporters located both in astrocytes and adrenergic neurons, one could expect efficient inhibition of both glial uptake and neuronal re-uptake of noradrenaline [29, 48].

Importantly, neither ATX nor EE were able to enhance the LTP in the old dnSNARE mice (Fig. 1c, d) strongly suggesting the key role of astroglial exocytosis in these effects. These observations closely agree with our previous results obtained in the neocortex [5, 22, 36, 49, 50, 52], which demonstrated that enhancement of astroglial adrenergic signalling and, consequently, release of ATP and D-Serine from astrocytes can counter-balance the age-related alterations in synaptic transmission and plasticity. Interestingly, ATX increased not only the astrocytic Ca^2+^-transients evoked by noradrenaline, but also the transients evoked by endocannabinoid receptor agonist anandamide (AEA) and Ca^2+^ responses to the synaptic stimulation (Fig. 1b). The latter effects might be explained by the interaction of astroglial adrenoreceptors with other G-protein coupled receptors [53], in particular trough regulation of their membrane trafficking via beta-arrestins [54, 55]. One have to emphasize that concentration of ATX used in our experiments was very small and could be achieved during daily oral administrations at 0.1–0.15 mg/kg.b.w. dosage, which is a several times than recommended dosage for human patients [56, 57].

These results highlight atomoxetine and, possibly, other selective NA re-uptake inhibitors as perspective exercise-mimetics whose beneficial effects in the ageing brain can, most likely, be mediated via enhancement of gliotransmission and astroglia-neuron interactions.

### Role of Autophagy in the Cognitive Effects of Caloric Restriction

Apart from physical exercise, caloric restriction (CR) usually defined as a reduced intake of calories not causing malnutrition, is widely suggested as an efficient strategy to improve health- and lifespan in most living organisms [9, 12, 13, 58]. Over the years, numerous studies have reported that CR can exert a variety of life-extending effects and counteract various age-associated biochemical alterations both in the experimental models and human patients [2, 9, 12, 13, 42, 58]. In mammals, including primates, CR was shown to have beneficial effects on longevity and promote an increased healthspan-the period of life during which an individual is health-by reducing the incidence of metabolic diseases, generally cancer, arteriosclerosis and neurodegeneration[12, 59].

Although a variety of beneficial effects of CR on brain health and cognitive healthspan have been reported so far [12, 59–61], they have been associated mainly with improvement of brain health via various metabolic and neuroendocrine [9, 12, 59] pathways. However, our recent work have demonstrated that CR can exert more subtle effects on synaptic transmission, plasticity and glia-neuron communications [5, 7]. We found that CR can alleviate age-related alterations in the synaptic transmission, in particular up-regulating the glutamatergic synaptic currents and down-regulating GABAergic inhibition in the neocortical pyramidal neurons [7, 28]. As a result, CR was able to rescue the long-term synaptic plasticity in neocortical neurons of old wild-type mice [5, 7]. Most likely, these beneficial effects are mediated by astrocytes because (1) CR enhanced astroglial Ca^2+^ signalling and release of gliotransmitters such as ATP and D-Serine [5, 28]; (2) beneficial effects of CR were strongly attenuated in the dnSNARE mice with selective impairment of astroglial exocytosis [5, 28]. These results suggest that astroglia can serve as a key cellular mediator of benefits of caloric restriction on cognitive functions.

It is widely suggested that life-extending effects and health benefits of caloric restriction originate mainly from the enhancement of autophagy [12, 62–64]. Macroautophagy (hereafter autophagy) is a cellular mechanism by which aggregated/misfolded proteins and damaged organelles are engulfed within double-membrane vesicles (autophagosomes) and then, after several stages of vesicular fusion and maturation, delivered to lysosomes for degradation [40]. Initiation of autophagy in mammalian cells occurs via the activity of the Beclin1—vacuolar sorting protein 34 complex (Beclin1-VPS34 complex) [42, 58, 65–67]. Upstream of Beclin1-VPS34 complex, initiation of autophagy is regulated by a range of different molecular pathways including highly conserved nutrient and energy sensors mTOR (mechanistic target of rapamycin) and AMP- activated kinase (AMPK). Autophagy can also be modulated in a bi-directional manner by lysine acetyltransferases (e.g.EP300) or deacetylases (e.g., sirtuins) which correspondingly inhibit or promote autophagy [13, 42, 62]. As a catabolic process, autophagy is very sensitive to the decrease in nutrients level so it could be readily activated by CR and fasting regimens. Indeed, CR was widely reported to modulate several key regulatory cascades of autophagy [9, 13, 60]. For instance, can CR activate AMPK, which serves as an energy sensor that inhibits the mTOR kinase activity (autophagy suppressor), thereby leading to autophagy induction. Also, CR can directly and indirectly activate sirtuins (SIRTs) causing protein deacetylation and enhancement of autophagy. SIRT1 and AMPK may engage in a positive feedforward loop thereby amplifying the response to CR [9, 13]. In a practical sense, CR has become a “method of choice” to induce autophagy in different types of experimental animals [9, 58].

Although molecular mechanisms underlying the role of autophagy in health and disease are not clear yet, beneficial effects of autophagy are commonly attributed to the digestion of potentially harmful intracellular structures, like aggregates of misfolded dysfunctional proteins and damaged organelles [9, 42, 66]. The cytoprotective and life-extending effects of autophagy have been observed in various experimental models, including human patients [9, 42, 62]. The capacity of a cell to autophagic degradation declines with age [13, 63, 66, 68] and alterations of autophagy have been implicated in many ageing-related pathological processes, including various neurodegenerative diseases [42, 63, 66]. On the another hand, chemical or genetic enhancement of autophagy has been reported to ameliorate signs of the neurodegenerative diseases in various animal models [58]. Recent data highlighted the importance of neuronal and glial autophagy for synaptic homeostasis and plasticity [66, 68–71]. In contrast to the recent advances in the study of roles for neuronal and microglial autophagy in neurodegeneration, relatively little is known about the specific mechanisms and (patho)physiological roles of autophagy in astrocytes. Since autophagy is an evolutionarily conserved process essential for cell vitality, there are strong grounds to believe that regulation of autophagy in astrocytes is mediated by similar molecular cascades as neuronal autophagy [72, 73]. Indeed, astrocytes have been reported to express a variety autophagy-related proteins and changes in expression of these proteins, in particular LC3, P62, Atg5, Atg7 and ULK2 have been linked to astroglial response to nutritional and oxidative stress and neuroinflammation [72, 73]. There is also emerging evidence of astroglia-specific changes in autophagy under certain pathological conditions [74, 75]. In particular, it has been reported that autophagy in astrocytes can be activated much strongly, as compared to neurons, by the metabolic stress and pharmacological inhibition of MTOR [74]. Still, role for cell type-specific changes in autophagy in brain aging and neurodegeneration is yet to be established.

One of the major challenges in the therapeutic applications of various CR protocols in human patients, like daily reduction in food intake or intermittent fasting, is that relatively long-term CR is required to produce beneficial effects on healthspan and lifespan, which undermines applicability of such interventions [13, 60]. Thus, development of pharmacological CR-mimetics which could induce the beneficial effects of CR without provoking diet-related discomfort attracts a great interest. It is no wonder that most perspective CR-mimetics have being sought among modulators of autophagy [9, 13, 64].

## Autophagy Modulators as CR-Mimetics

One of “oldest” classes of CR-mimetics is *rapalogs* represented by mTOR inhibitor rapamycin (sirolimus) and its analogs, such as temsirolimus and everolimus. Due to the presence of multiple molecular targets of mTOR, rapalogs were reported to have a variety effects on cellular metabolism and senescence majority of which converge to suppression of gene transcription and protein synthesis and induction of autophagy and, consequently, reduction of protein aggregation[9, 60, 64, 76]. This anti-aggregation action of rapalogs, most likely, explains their neuroprotective effects reported in several models of [9, 60, 76, 77] neurodegenerative diseases. Since life-extending properties of rapalogs have been reported in numerous studies from yeast to mammals, series of clinical trials for specific clinical applications have been conducted or registered for some of these drugs but the obtained results, especially on health- and/or lifespan-extending effects, were not entirely conclusive [9, 63, 76]. Furthermore, recently emerged data suggest that inhibition of mTOR by rapamycin can also cause negative effects on astroglia functions. For instance, inhibition of mTORC1 has been reported to activate astrogliosis [78], impair the development of the dentate gyrus by depleting the astroglia and microglial cellular pools [79] and inhibit release of purine gliotransmitters from spinal cord astrocytes [80]. These results cast some doubts upon usefulness of rapalogs as “enhancers” of astroglia-neuron interactions in ageing brain.

**Fig. 2:**
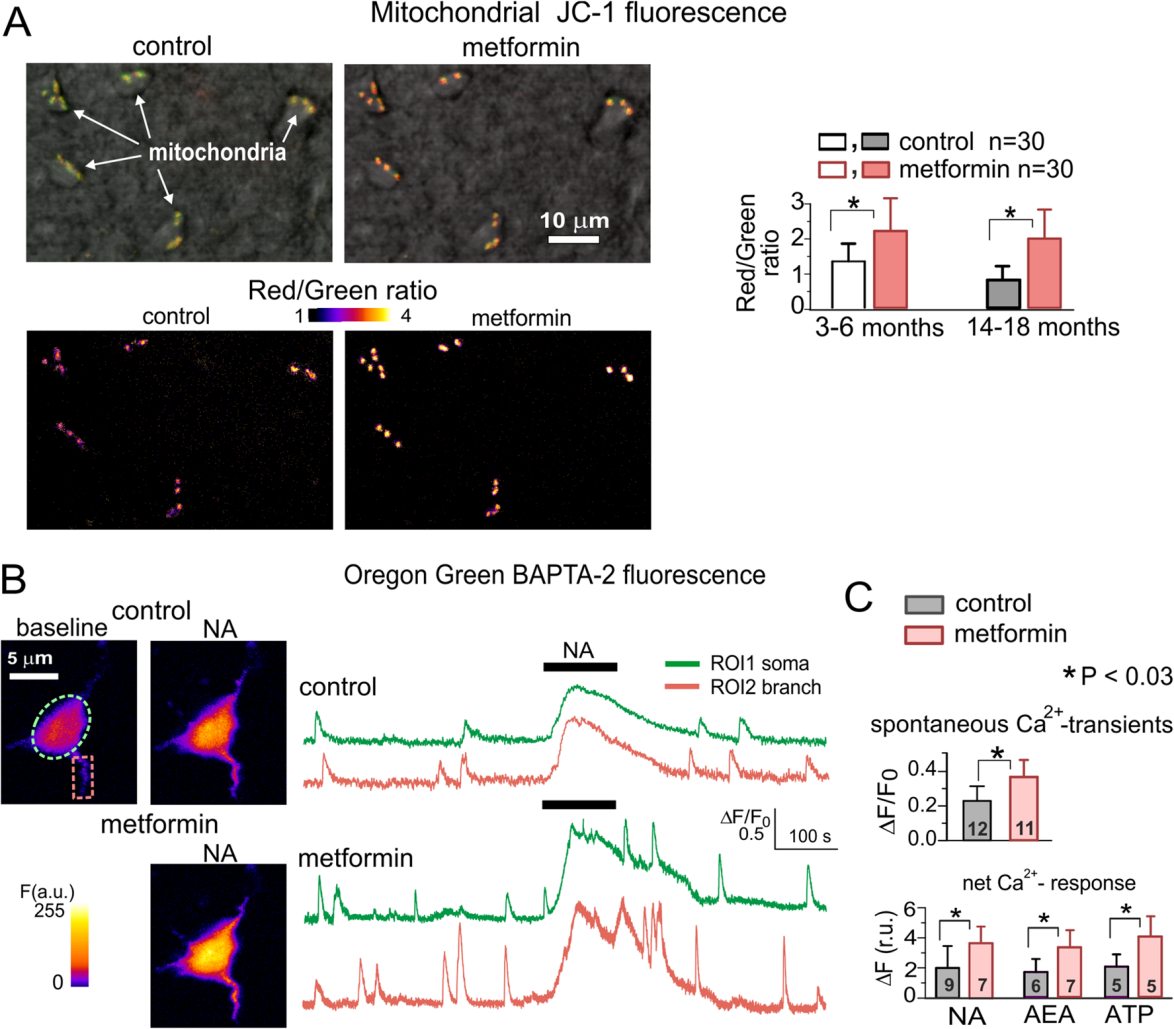
Impact of metformin on the mitochondrial potential and Ca^2+^−signalling in neocortical astrocytes. **a** Mitochondrial potential was evaluated with the aid of mitochondria-specific potential-sensitive ratiometric dye JC1 (*Molecular Probes*). The ratio of red to green fluorescence of JC-1 is dependent only on the mitochondrial membrane potential and decreases with mitochondrial depolarization. Neocortical slices were preloaded with 2 µM of JC-1 for 40 min and green (540 ± 15 nm) and red (630 ± 35 nm) fluorescence was measured before and after 20 min after application of 5 µM metformin using Zeiss LSM-7 2-photon microscope (excitation at 810 nM). Astrocytes were initially identified by their morphology under gradient-contrast illumination, their identification was confirmed at the end of recordings by electrophysiological characterization [25]. The upper images show superposition of the gradient-contrast image of astrocytes with red and green JC-1 fluorescence in the control and after incubation with metformin, the lower pseudo-color images show corresponding ratio of red to green signal. The diagram shows pooled data (mean ± SD for 30 cells) on the JC-1 fluorescence recorded in astrocytes from young (3–6 months) and old (14–18) mice. Note the age-related decrease in the red/green ratio, indicative for mitochondrial depolarization, in the old mice and metformin-induced increase in the mitochondrial potential. **b**, **c** 2-photon fluorescent images (pseudo color) and Ca^2+^-signals were recorded in the neocortical astrocytes of 14 months- old mice loaded with Ca^2+^-indicator OGB-2AM, as described previously [5, 22, 25], before (control) and 20 min after application of 5 µM metformin. **c** The pooled data on the amplitude of spontaneous Ca^2+^ and net response to 1µM noradrenaline (NA, 1 µM) and CB1 receptor agonist anandamide (AEA, 500 nM). Note the significant increase both in the spontaneous and evoked Ca^2+^-signalling after the application of metformin.

Another relatively well-studied CR-mimetic is metformin (dimethylbiguanide hydrochloride) which was extensively used as anti-diabetic drug [81]. Various metabolism-related anti-aging effects of metformin were observed in animal models and human patients, mostly in the context of age-related and neurodegenerative diseases where diabetes is a confounding factor [9, 81]. In particular, metformin has been reported to improve spatial and recognition memory in the aged rodents [82, 83] and regulate gene expression specifically in reactive astrocytes in the mouse model of Parkinson’s disease [84]. The beneficial effects of metformin have been attributed to the several molecular cascades, mainly to the inhibition of the mitochondrial electron transport chain complex I and activation of AMPK, inhibition of mTORC1 independently of AMPK and direct regulation of SIRT1 [9, 81]. Apart from regulation of autophagy, metformin can have pleiotropic anti-aging effects, the most important of which two in the context of present review is improvement of mitochondria function via modulation of oxidative phosphorylation and relieving the oxidative stress [9, 81]. Since mitochondrial play important role in the regulation of cellular Ca^2+^-homeostasis, one might expect a beneficial effects of metformin on Ca^2+^-signalling in astrocytes. Indeed, we observed that incubation of neocortical slices of the old mice with 5 µM metformin increased the mitochondrial potential and enhanced adrenergic Ca^2+^-signalling in astrocytes (Fig. 2), thus ameliorating age-related decline in astroglial function. Taking into account the positive effect of metformin on Ca^2+^-dynamics in astrocytes one might suggest to combine it with adrenergic EE-mimetics, such as atomoxetine. As added benefit of such combination, metformin could alleviate the most plausible side-effect of NA-uptake inhibitors – hypertension. The anti-hypertensive action of metformin is rather well-documented, it relies on the ability of metformin to promote mitochondrial biogenesis in vascular endothelial cells and activate the endothelial NO synthase and NO-dependent vasodilation via AMPK-dependent pathway [81].

**Fig. 3:**
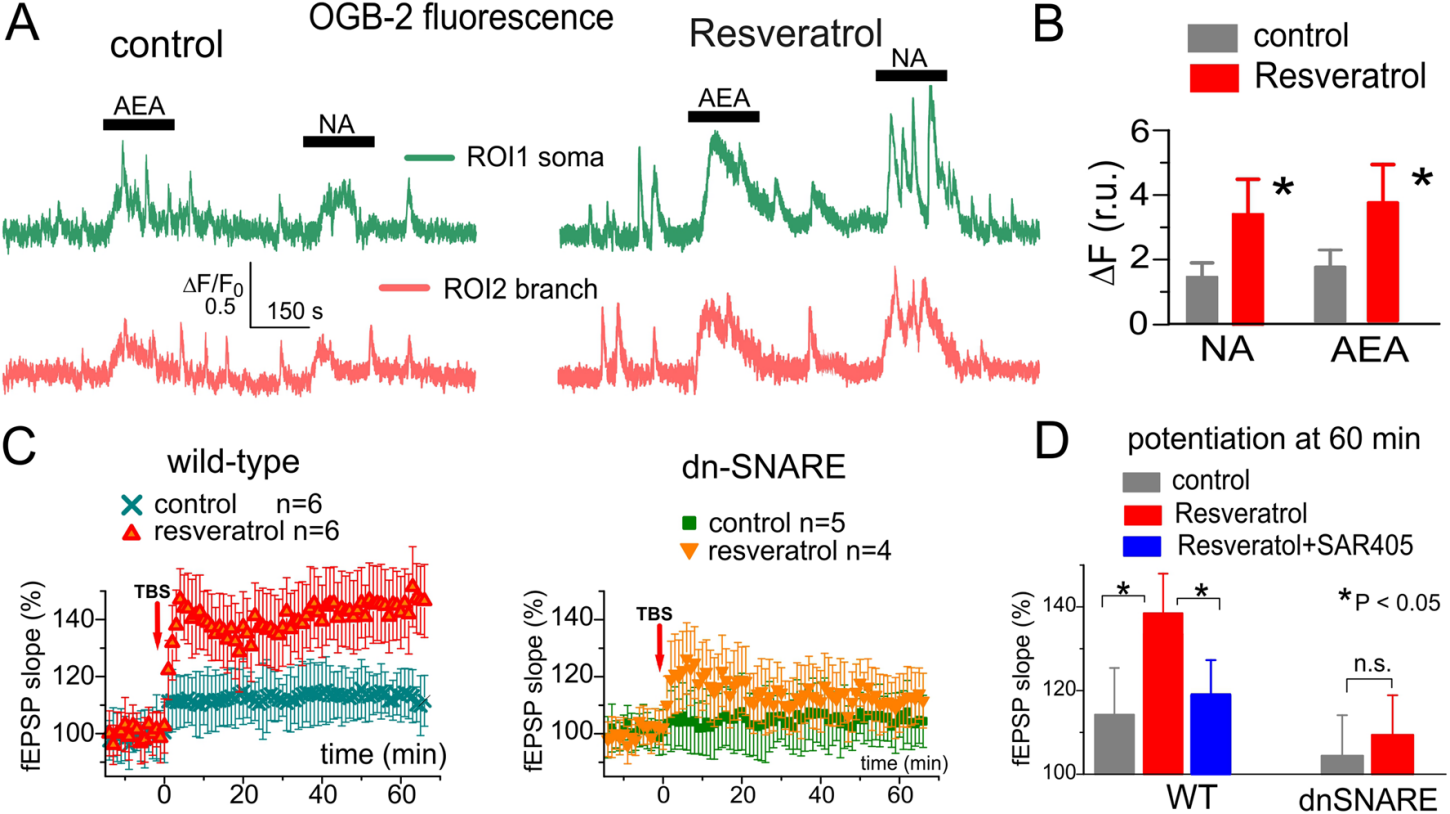
Effects of resveratrol on astroglial Ca^2+^ signalling and synaptic plasticity in neocortex of old mice. **a**, **b** Ca^2+^-signalling was evaluated in the astrocytes of layer 2/3 of somatosensory cortex of 12–16 months old wild-type mice as described in [5, 22, 23]. Astrocytic Ca^2+^-transients were activated by agonists of CB1 receptors (AEA 500 nM) and adrenoreceptors (NA, 1 µM) in control and after 1 h-long pre-incubation with resveratrol (20 µM). **a** Representative 2-photon recordings of OGB-2 fluorescence in the neocortical astrocytes of 13 months-old wild-type mouse. **b** Pooled data for n = 10 astrocytes from 12 to 16 old mice. **c**, **d** The long-term potentiation of the field EPSPs in the neocortical layer 2/3 of 12–16 months old wild-type and dnSNARE mice. The LTP was induced by 50 pulses of theta-burst stimulation (as described in [5]) in the control and after 1 h pre-incubation of slices with resveratrol alone or resveratrol in combination with autophagy inhibitor SAR405 (200 nM). **d** Pooled data on the magnitude of LTP at 60 min, the data are shown as mean ± SD for the number of experiments indicated in (**c**). The beneficial effect of resveratrol is significantly reduced in the dn-SNARE mice with impaired exocytosis of gliotransmitters from astrocytes and is antagonized by selective inhibitor of autophagy SAR405

Third group of perspective CR-mimetics are polyphenols abundant in the skin of grapes and in the red wine, in particular *resveratrol* [13, 60, 85]. Resveratrol has been shown to promote longevity across species, in particular ameliorate the age-related changes in metabolic and physiological parameters and extend life-span of mice on a high-fat diet. Also, resveratrol induces a CR-like transcriptional signature in mice and recapitulates metabolic metabolic changes of CR in humans [9, 68, 85]. Similarly to metformin, resveratrol targets a number of stress-related cellular components, including the AMPK and SIRT1 which in turn can regulate autophagy [9, 68, 85]. Moreover, the resveratrol-induced increase in lifespan was abolished following knockdown of essential autophagy genes, such as *Beclin1 *[9, 68]. As perspective autophagy modulator, resveratrol is gaining increasing interest as potential drug for treatment of neurodegenerative disorders [44, 63, 86]. Still, the molecular and cellular mechanisms underlying plausible anti-aging effects of resveratrol on the function of aging brain remain elusive. Although the health-promoting and life-extending effects of resveratrol are presumed to be mediated by modulation of autophagy [9, 85], resveratrol has been shown to produce significant effects on AMPK and mTOR only when applied in super-pharmacological concentrations (> 20 µM)[87, 88]. In this regard, our preliminary data demonstrating that moderate concentrations (10 µM in situ) of resveratrol can enhance the spontaneous and evoked and astrocytic Ca^2+^-signalling and synaptic plasticity (Fig. 3) attract a particular interest. The effect of resveratrol on the synaptic plasticity was partially reduced by the autophagy inhibitor SAR405 (Fig. 3d) and was precluded in the dnSNARE mice with impaired astroglial exocytosis. Hence, one could suggest that resveratrol in moderate doses acts via enhancement of astroglial Ca^2+^-signalling and glia-neuron interactions which might involve regulation of neuronal autophagy.

Recently, a naturally occurring polyamine spermidine has emerged as promising anti-aging agent [13, 60, 89]. Spermidine can readily interact with negatively charged molecules, including DNA, RNA and lipids, thereby affecting basic cellular functions such as DNA stability, as well as cell growth, proliferation and, importantly, autophagy [62]. Spermidine can cause both a rapid induction and sustained enhancement of autophagy, correspondingly through the inhibition of the acetyl transferase EP300, resulting in deacetylation of autophagy-relevant cytosolic proteins, and inhibition of histone acetyl transferases, leading to the induction of autophagy-relevant gene transcription [13, 62]. Spermidine can be obtained from food or synthesized in mammalian tissues so it can serve as a endogenous and exogenous modulator of autophagy. Due to this dual role, health-promoting and life-extending effects of spermidine attracted a great interest both in the fundamental and clinical studies [9, 62]. The blood concentration of spermidine has been reported to undergo age-related decrease and inversely correlate with severity of symptoms of age-related diseases and mortality rate [13, 62, 63, 69].

**Fig. 4:**
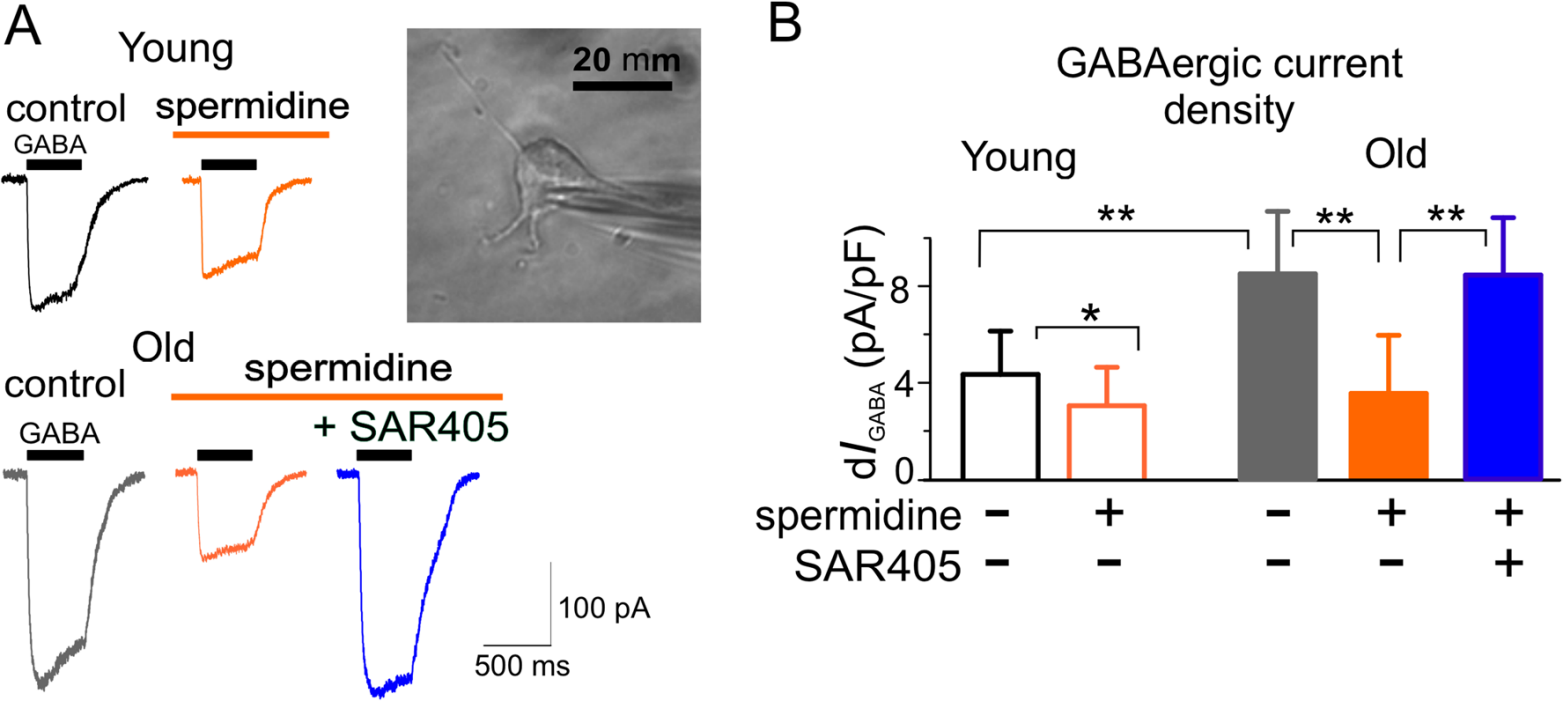
Spermidine down-regulates GABAergic inhibitory currents in the neocortical neurons. **a** The GABAergic currents were evoked in the acutely isolated neurons of 3 months-old (young) and 14 month-old (old) mice by application of 100 µM GABA and measured using whole-cell voltage-clamp as described previously [25, 50, 52]. Neurons were acutely dissociated from the hippocampal slices [50, 52] incubated either with spermidine (10 µM) alone or with spermidine and autophagy inhibitor SAR405 [86] (200 nM), which specifically inhibits vacuolar sorting protein VSP34. **b** Pooled data on the density of GABA-induced currents under different conditions (mean ± SD for 5 cells). Note that spermidine causes significant down-regulation of GABAergic currents which is blocked by SAR405, suggesting an involvement of autophagic mechanisms

So far, majority of the data on beneficial effects of spermidine on synaptic plasticity has been obtained in flies [62, 66, 69] and mechanisms of its action on mammalian brain cells are not well-established. Our experiments in the neocortical neurons of aged mice (Fig. 4) show that spermidine can up-regulate neuronal autophagy and down-regulate the GABAergic transmembrane currents in the autophagy-dependent manner (Fig. 4c). The latter effect of spermidine could, potentially, shift the balance between excitation and inhibition and thereby facilitate the induction of long-term synaptic plasticity. Still, influence of spermidine on astroglial signalling and glia-neuron interaction remain to be explored.

### Effects of Autophagy Modulation in the Context of Synaptic Transmission and Astrocyte–Neuron Interactions

Majority of the effects of autophagy on brain function reported so far has been linked to retarding neurodegeneration via improvement of neuronal metabolism and proteostasis [12, 42, 66]. Nevertheless, there is growing evidence that molecular machinery of autophagy can be directly involved in regulation of synaptic transmission and plasticity, affecting the synaptic efficacy both at presynaptic and postsynaptic *loci* [68, 71]. These effects could originate from interaction of autophagy-regulating proteins with mechanisms of trafficking of synaptic vesicles and membrane trafficking, endocytosis and degradation of neurotransmitter receptors and ion channels [67, 68, 71]. One might argue that in ageing brain direct impact of autophagy on synaptic dynamics could be no less important than the autophagy-mediated neuroprotection. Recent data obtained in the model organisms (flies) suggest that decline in autophagic capacity of neurons can cause synaptic degeneration and cognitive deficits [66, 70]. At the same time, enhancement of autophagy by dietary spermidine has been reported to ameliorate the age-induced memory impairment, most likely via autophagy-dependent rejuvenation of synaptic active zones [58, 62].

The specific molecular cascades underlying putative role for autophagy in the effects of CR and CR-mimetics on synaptic transmission and cognitive functions remain largely unexplored. Whereas numerous studies have reported various molecular interactions between autophagy-related proteins and elements of synaptic machinery, predominantly in non-mammalian models [42, 66, 68], observations of direct effects of modulation of autophagy on synaptic plasticity in mammalian brain tissue remain scarce [66, 71].

The less ambiguous is mechanism of autophagy-mediated modulation of inhibitory synaptic transmission which, most likely, involves the modulation of GABAA receptor trafficking via Beclin1-VSP34-GABARAP cascade [86]. This mechanism has recently been implicated in the effects of VSP34 inhibitor SAR405 on inhibitory neurotransmission in amygdala and fear memory [86]. Our observations of the action of SAR405 on synaptic plasticity in neocortex (Fig. 3) and down-regulation of GABAergic currents by spermidine (Fig. 4) strongly support the notion that autophagy regulates inhibitory synaptic transmission via Beclin1-VSP34 cascade.

The impact of autophagy on glutamatergic excitatory transmission remains uncertain, especially at the post-synaptic site [66, 71] where, arguably, most important events of long-term synaptic plasticity occur. On the one hand, the impact of autophagy on recycling endocytosis and lysosomal sorting of glutamate receptors has recently been suggested as important regulator of strength of excitatory synapses [71, 90, 91] and on the another hand, different studies attributed the improvement of synaptic plasticity (in different pathological context) both to the decrease and increase in autophagy (in the different pathological context) [9, 42, 66, 68, 71]. Two main molecular pathways has been suggested to be responsible for regulation autophagy in excitatory synapses: down-regulation of autophagy via regulation of mTOR by BDNF and enhancement of autophagy by spermidine acting via protein de-acetylation and FOXO transcription factor [9, 66, 71].

Intriguingly, astrocytes can participate in the storage and release of the above-mentioned endogenous modulators of autophagy. Astrocytes have been reported to release BDNF [27] and astroglia-derived BDNF has been implicated in the homeostatic scaling of glutamatergic synapses during EE [28]. Although the latter effect has been attributed to the TrkB receptor-MSK1-Arc pathway [28, 92], the modulation of autophagy by astroglia-derived BDNF is still plausible. As for the spermidine, astrocytes have high capacity for uptake of spermidine form the blood by virtue of expressing the organic cation transporters OCT1-3 [93] and its content in astrocytes was reported to be much higher than in neurons [94, 95]. Finally, it has been recently discovered that astrocytes can store spermidine in vesicles by virtue of abundant expression of vesicular polyamine transporter *SLC18B1 *[96].

**Fig. 5:**
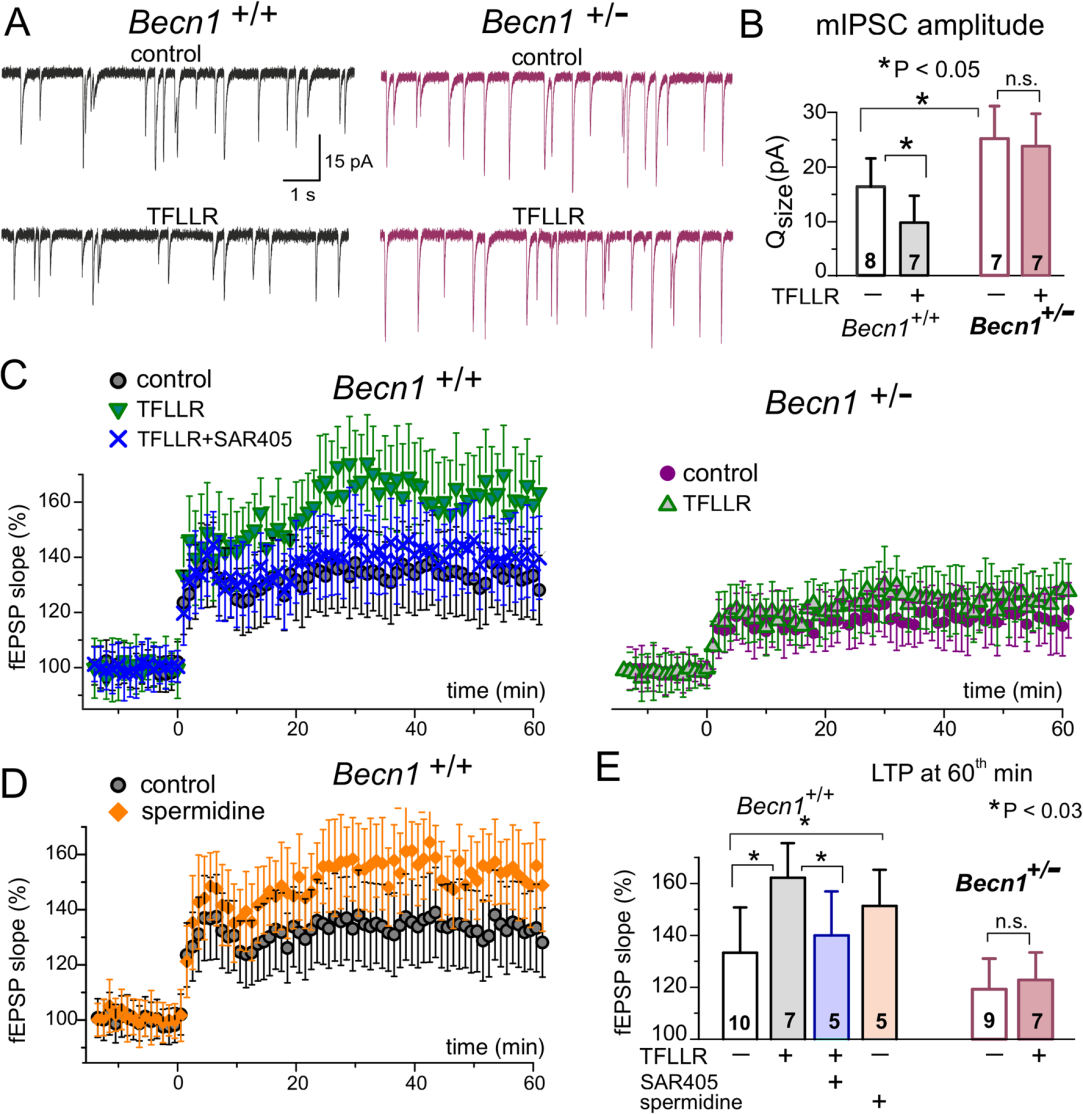
Impact of activation of astrocytes on the inhibitory synaptic transmission and synaptic plasticity in the neocortical neurons of autophagy-deficient mice. The Beclin1-deficient mice (*Becn1*^+/−^) were obtained by crossing the Becn1^fl/fl^ and Camk2a^creERT2/+^ transgenic lines. The 6–8 weeks old mice were kept under standard housing conditions for 4 weeks after tamoxifen injections. **a**, **b** The miniature spontaneous inhibitory synaptic currents (mIPSCs) recorded in the neocortical layer 2/3 pyramidal neurons of the *Becn1*^+/−^ mice and their wild-type littermates (*Becn1*^+/+^). **a** The representative mIPSCs were recorded as described in [25] in the presence of NBQX and TTX in the control conditions and after exposure of hippocampal slices to the agonist of astroglia-specific PAR1 receptors TFLLR (3 µM). The quantal amplitude and frequency of the mIPSCs were quantified as described in [25, 52]. **b** The diagram shows the pooled data (mean ± SD for number of neurons indicated) on the changes in quantal amplitude of mIPSCs (indicative for the postsynaptic changes) induced by activation of astrocytes with TFLLR. Note the increase in the amplitude of baseline mIPSCs and the lack of effect of TFLLR in the *Becn1*^+/−^ mice. **c**-**e** The long-term potentiation of fEPSPs in the neocortical layer 2/3 of the same mice cohorts as in (**a**, **b**). The LTP was induced by 30 pulses of theta-burst stimulation (as described in [5]) in the control and after pre-incubation of slices with TFLLR alone or TFLLR with 200 nM SAR405 **c** or with 10 µM spermidine (**d**). **e** Pooled data on the magnitude of LTP in the wild-type and *Becn1*^+/−^ mice (mean ± SD for the number of experiments indicated). Note the impairment of LTP in the *Becn1*^+/−^ mice and inhibition of the TFLLR-induced facilitation of LTP by autophagy inhibitor SAR405

Thus, one might speculate that astrocytes can regulate neuronal autophagy by releasing BDNF and/or spermidine. However surprising and fanciful this hypothesis may seem, it is supported by our pilot data on astroglia-driven modulation of synaptic transmission and plasticity in the neocortical neurons of the Beclin1-deficient mice (Fig. 5). As a model of inducible impairment of neuronal autophagy, we used the cross between Camk2a^CreERT2/+^ and Becn1^Fl/Fl^ [97] lines (Becn1^+/−^ mice). We evaluated the miniature spontaneous inhibitory currents (mIPSCs) in the pyramidal neurons and long-term synaptic potentiation of field EPSPs in the neocortical layer 2/4. For sustained tonic activation of astrocytes, neocortical slices were incubated for 1 hrs with 3 µM of PAR-1 receptor agonists TFLLR (followed by 30 min wash-out), this approach has been previously demonstrated to activate Ca^2+^-signalling in selectively in astrocytes and trigger the release of gliotransmitters [25, 34, 52]. To avoid a plausible effect of astroglia-derived ATP on the GABAA receptors [25], TFLLR was applied in the presence of purinoreceptors antagonists PPADS and 5-BDBD. Under control conditions, the average amplitude and quantal size of the GABAergic mIPSCs in the neurons of Becn1^+/−^ mice were significantly larger (Fig. 5a) and the magnitude of LTP significantly smaller (Fig. 5c) than in their wild-type (Becn1^+/+^) littermates. Activation of astrocytes with TFLLR considerably decreased the mIPSCs in the wild-type but not in the Beclin1-deficient mice (Fig. 5a, b). In the wild-type mice, the effect of astrocyte activation on mIPSCs was reproduced by the preapplication with 10 µM of spermidine and decreased by the VSP34 inhibitor SAR405 (Fig. 5b). In line with the changes in the inhibitory synaptic currents, incubation of neocortical slices with TFLLR or spermidine enhanced the LTP in the neocortex in the VSP-34 and Beclin1-dependent manner (Fig. 5c, d). These results may imply that astrocytes can release some factors, presumably spermidine, to regulate neuronal autophagy at the post-synaptic locus, most likely via the Beclin1-VSP34-pathway.

### Future Directions

It becomes evident that caloric restriction and its pharmacological mimetics have a great therapeutic potential for treatment age-related brain disorders. Taken together, recent data indicate that astroglia can have a pivotal role in the beneficial effects of exercise and caloric restriction and their pharmacological mimetics on brain health and cognitive longevity. Apart from improvement of metabolism and proteostasis in the neuronal and glial cells, exercise- and caloric restriction-mimetics can exert more subtle, but not less important, effects on synaptic transmission, in which astrocyte can take an active role. The most plausible mechanism underlying such a role is enhancement of astroglial Ca^2+^-signalling and release of gliotransmitters which, in turn, modulate synaptic dynamics and homeostasis thereby alleviating the age-related decline in synaptic plasticity and cognitive functions. A good example of such action is provided by the perspective exercise-mimetics atomoxetine, the noradrenaline uptake inhibitor. Its positive effect on astroglial signalling might be synergistically enhanced by CR-mimetics metformin which can act via improvement of mitochondrial function. Still, the suitability of atomoxetine or atomoxetine/metformin combination for treatment of aging-related cognitive decline is yet to be investigated.

A variety of positive effects of many CR-mimetics, such as metformin or resveratrol, on synaptic transmission and plasticity in the aging brain involve regulation of autophagy and the most intriguing question here is whether astroglia can modulate neuronal autophagy by releasing some endogenous modulators, presumably spermidine or BDNF. However, the data on impact of CR-mimetics and autophagy modulators on synaptic function and astrocyte-neuron interactions in the aging mammalian brain are very scarce and their efficiency in preventing age-related cognitive decline is yet to be fully explored, in particular in the mammalian animal models of cell-type specific impairment of autophagy.
